# The Role of Social Ties and Social Support in Maintaining Physical and Mental Well-Being Among Korean Americans Living with Chronic Hepatitis B

**DOI:** 10.1007/s40615-025-02819-6

**Published:** 2026-02-02

**Authors:** Eunji Kim, HeeSoon Juon, Giyoung Lee, Katherine C. Smith, Mimi Chang, Ann C. Klassen

**Affiliations:** 1 Department of Community Health and Prevention, Drexel University Dornsife School of Public Health, Philadelphia, PA 19104, USA; 2 Department of Medical Oncology, Thomas Jefferson University, Philadelphia, PA 19107, USA; 3 Department of Health, Behavior and Society, Johns Hopkins Bloomberg School of Public Health, Baltimore, MD 21205, USA; 4 Asian Pacific Liver Center, Coalition of Inclusive Medicine, Los Angeles, CA 90020, USA

**Keywords:** Hepatitis b, Asian American, Immigrant, Social support, Social ties, Chronic diseases

## Abstract

Chronic Hepatitis B (CHB), a serious disease requiring lifelong management, disproportionately affects Asian Americans, including Korean Americans. Many Korean Americans experience linguistic and sociocultural barriers, potentially worsening health challenges. While social ties and social support are well-recognized determinants of health, their role within unique populations merits exploration. Using longitudinal survey data, we identified patterns of social ties and social support among Korean Americans living with CHB, and how these related to self-assessed physical and mental health over time. Patients in Los Angeles and Philadelphia (*n* = 309) completed surveys at two timepoints between 2021 and 2024. We measured social ties with the Lubben Social Network Scale (LSNS-6), social support with the eight-item Medical Outcomes Study Social Support Survey (mMOS-SS), and self-assessed health using SF-12 Physical and Mental Component Summary Scores (PCS, MCS). Greater baseline social ties were higher among married, college-educated, and bicultural- or Western-identifying participants. Social support scores were higher among married, English-fluent, and currently working participants. Greater social ties were associated with better baseline physical health (β = 0.134, *p* = 0.009), and greater social support with better mental well-being (β = 0.206, *p* < 0.001). Controlling on baseline health, social ties predicted better follow-up PCS (β = 0.108, *p* = 0.012), and social support predicted better follow-up MCS (β = 0.136, *p* = 0.007), suggesting that social ties and social support independently and differentially influence physical and mental health over time. Strengthening both structural connections through social ties and perceived social support may delay health decline among immigrant populations managing chronic conditions.

## Burden of Chronic Hepatitis B Infection

Hepatitis B infection is a significant global health issue, and the most common serious viral liver infection [1, 2]. Chronic hepatitis B (CHB) can lead to severe disease, including cirrhosis and liver cancer, and can ultimately result in death [3]. Individuals living with CHB face the need for continuous health monitoring throughout their lives [4–6].

Asia has the highest burden of Hepatitis B Virus (HBV) infection [5]. The population of Asian Americans has been growing rapidly in the United States, with 70% of Asian Americans born outside the U.S [1, 2]. Despite being only 6% of the U.S. population, Asian Americans make up 58% of the 862,000 Americans with diagnosed CHB [1, 2]. The prevalence of CHB is 31 times higher among non-Hispanic Asians (3.41%) than whites (0.11%) [7].

Asian populations continue to have a disproportionately high prevalence of HBV, with widespread infection caused by previous lack of universal immunization as well as vaccination at birth to prevent mother-to-child transmission [8]. Although immunization programs have dramatically reduced new infections, millions of adults remain chronically infected. Recent global modeling efforts estimate an overall worldwide HBV prevalence of 3.2% in 2022, with substantially higher levels in the Western Pacific (7.1%) and parts of Southeast Asia (3.8%), reflecting historical transmission patterns and persistent variation in timely birth-dose coverage [8].

In South Korea, despite nearly universal infant immunization and elimination of perinatal transmission, the overall prevalence of HBV remains approximately 2.7% [8], primarily due to infections contracted prior to universal infant vaccination in 1995 [8, 9]. A significant portion of Korean adults who have immigrated to the U.S. belong to earlier birth cohorts, contributing to higher rates of chronic HBV among Korean American immigrants.

### The Role of Social Relationships in CHB Management

Social relationships are essential factors in the physical and mental health of Asian Americans. Especially for older immigrants, social relationships, particularly familial ties, play an essential role in preserving cultural values and customs [9, 10]. When living in host countries among individuals who do not share their primary language and culture, the family’s central function may persist or even become more significant [9, 10]. Previous studies have demonstrated that support from family members, friends, and community members helps people with chronic illnesses better manage their conditions and feel less isolated, leading to better health outcomes [11, 12]. Healthcare service utilization and health outcomes of elderly Asian immigrants, regardless of their English proficiency and health literacy, can improve substantially with social support from spouses, friends and neighbors and adult children [13].

For Korean Americans specifically, challenges associated with CHB are heightened by their predominantly foreign-born status [14]. Korean immigrants face heightened burden managing CHB in the U.S. due to language barriers, immigration status, and disadvantaged socioeconomic conditions [15]. After relocating to a new country, they face challenges in communication, employment, and health care and service access, all of which can adversely impact their physical and mental health [16, 17]. Previous studies have proven that foreign-born Korean Americans experience greater social isolation because they have limited English proficiency [18], struggle with cultural adaptation [19], and experience disrupted support systems post immigration [20]. Changes in social life, such as fewer close family and friends, can lead to isolation and loneliness, potentially resulting in harmful coping habits [11, 13]. Moreover, the support system enhances physical activity while decreasing loneliness, which leads to better subjective well-being and quality of life for Korean older adults [21]. Given the strong emphasis on familism and interdependence in Korean culture [18], the absence of social connections with family and friends makes them more vulnerable to negative impacts on their physical and mental well-being [20, 22].

Since mother-to-child transmission of HBV is a major route of HBV spread in many Asian regions [4, 5, 7], for Korean Americans with CHB, managing the vertical transmission of HBV from parents to children, along with lifelong treatment and monitoring [4, 5], can be improved through supportive relationships with their immediate family members or close social ties. Close relationships serve as a foundation for patients to follow their medication plans, maintain consistent clinic appointments and handle their disease properly.

However, HBV-related stigma and its impact on family and social relationships can burden Korean patients with CHB. Research shows that CHB patients endure widespread discrimination and stigma because of their diagnosis [23, 24]. Stigma was consistently observed in research involving individuals with chronic HBV, with many studies indicating that individuals often had feelings of embarrassment or shame, or that having HBV caused disgrace to their family [23, 25, 26]. The challenges patients face when disclosing their condition and seeking help outside their family circle may lead to social withdrawal and isolation, negatively impacting their physical and mental health and potentially creating additional health burdens.

### Theoretical Frameworks for Understanding Social Ties and Social Support

Social ties emphasize the importance of the structure and quality of an individual’s social relationships, consisting of family, friends, and community connections that can provide a framework for social support [27]. Social ties are often assessed by the type, closeness, and frequency of contacts, representing opportunities for social connectedness, and identifying individuals at risk for isolation. Social support, in contrast, is a subjective assessment by individuals of the emotional, informational, and tangible support they can call upon [28]. Importantly, social ties and social support are interdependent; social ties create the opportunity for, but do not guarantee social support.

Social support plays a critical role in an individual’s health and well-being [28]. One study of Chinese older persons found that having stronger and stable social support, (coupled with lower levels of social strain from challenging relationships), was associated with better mental health outcomes [29]. Additionally, research shows that having larger and more supportive social networks is associated with reduced feelings of isolation and improved overall health outcomes including fewer chronic conditions and less functional disability [30].

Social ties and social support are distinct elements of social connections, and are theorized to maintain health in different ways. The structure of social ties between people enables them to participate in social activities while sharing information and learning from others which promotes both healthy behaviors and consistent use of medical care [28, 31]. On the other hand, perceived social support functions as an emotional and psychological resource which helps people manage stress better and protects them from depressive symptoms [28, 32]. These distinctions are particularly relevant in chronic disease contexts, where in two forms which help patients with chronic disease management by enabling them to stick to their treatment plans and cope with illness-related stress through emotional support.

Studies to date have produced varied findings regarding the influence of social ties on the health of Asian immigrants. One study suggested that older Korean immigrants benefit more from non-family networks, such as friendships, than from kin-based family ties [33]. Friendships, along with other non-family relationships, led people to seek help more often while maintaining better mental health [33]. Another study involving older Chinese immigrants who relocated to the U.S. later in life showed that they experienced more depressive symptoms, attributed in part to lower income, poor health, and weak social connections [34]. Although social ties and support are generally important for good mental health, different racial and ethnic groups have varied approaches to utilizing social networks, and the effectiveness of social ties can be influenced by cultural preferences and the specific needs of immigrants [35, 36]. While social ties and support are generally associated with better physical and mental health outcomes, their roles among CHB patients are understudied and likely influenced by cultural and disease-specific characteristics.

The social convoy model, developed by Antonucci and colleagues, conceptualizes social resources as dynamic structures that evolve over time and provide necessary resources across the life course [37, 38]. This model conceptualizes social relations as convoys that accompany individuals throughout their life course, varying in size, structure, function, and quality based on personal factors (e.g., age, gender, culture) and situational factors (e.g., illness, stress) [37, 39]. Importantly, it highlights that the composition and contact frequency of close family and friend networks are fundamental indicators of social convoy structure, directly influencing health and well-being outcomes [37, 39]. The convoy model was originally conceptualized to explore changes across the time during aging. However, it can also offer an appropriate framework for understanding the impact of social resources on physical and emotional health within the context of the trajectories experienced over time as part of both immigration and chronic disease life journeys.

### Contribution of this Research

This research explores the role of social ties and social support in the physical and mental health of Asian Americans living with CHB in the United States, drawing on data collected as part of our longitudinal cohort study of Korean American patients at two clinical care settings, in Philadelphia and Los Angeles [40]. By exploring one specific population, the goal is to add to the diversity of evidence about this important social influence, and how it is understood across cultures and life situations. The analysis first examines the relationship between sociodemographic and immigration factors with social ties and social support, to describe how social ties and support vary among Korean Americans with CHB. Secondly, it examines the degree to which social ties and social support are associated with self-assessed physical and mental health functioning, both cross-sectionally and over time. Drawing on the convoy model [37, 38], we aimed to assess both the structural (network size and types) and functional (supportive interactions) dimensions of participants’ social relationships [37, 39].

Based on the literature and the social convoy model, our analysis distinguishes between social ties and social support. In addition, we focus on ties related to closer interpersonal relationships (such as with family members and friends), arguably more appropriate sources of support when living with potentially stigmatized illnesses like CHB, rather than the “looser” less intimate ties created through employment, church or more public relationships.

## Methods

### Study Design, Setting and Participant Sample

This longitudinal analysis uses survey data collected at two time points, as part of a larger prospective longitudinal cohort study, exploring bio-psycho-social factors that affect adverse health outcomes among Korean Americans with CHB [40]. Patients receiving care from bilingual Korean American hepatologists were recruited from two clinical sites in Philadelphia and Los Angeles. Eligible patients were identified through medical record reviews and invited to enroll during clinic visits between August 2021 and January 2023, and complete baseline surveys. Follow-up surveys occurred approximately two years post-enrollment (August 2023 to August 2024). Eligibility criteria included being age 18 or older, having no diagnosis of hepatocellular carcinoma (HCC), or another viral infection (e.g., HCV, HIV), and the ability to provide informed consent. Participants could choose to complete the electronic survey in English or Korean, with assistance as needed. All research components were reviewed and approved by the Institutional Review Board at Thomas Jefferson University. Participants provided written informed consent and received $50 for baseline and $25 for follow-up surveys.

### Independent Measures and Covariates

#### Social Ties and Social Support

Social ties were measured with six items adapted from the Berkman–Syme Social Network Index (SNI) [41] and the Lubben Social Network Scale (LSNS-6) [42]. These items assessed both the size and frequency of interactions within three types of social ties: living children, close relatives, and close friends. For each domain, participants reported the number of individuals in each category and the number seen at least monthly. Responses for each group were scored from 0 (none) to 1 (some but no monthly contact) to 2 (at least monthly contact). and summed them to create an index ranging from 0 to 6, with higher scores reflecting greater social connections. Cronbach’s α for the index was 0.66, indicating moderate internal consistency. Prior research has used a similar approach to assess family social ties among older Korean Americans [18, 43, 44].

Social support was measured using a modified eight-item version of the Medical Outcomes Study Social Support (mMOS-SS) scale, measuring key types of social support, ranging from instrumental to emotional: (a) help if confined to bed, (b) take to the doctors, (c) prepare meals, (d) help with daily chores, (e) have a good time with, (f) advice on dealing with an issue, (g) understanding your problems, and (h) providing love/feeling wanted [45], with respondents choosing from 5 categories of support availability (none of the time/rarely/sometimes/a lot of the time/all of the time). Possible summed scores ranged from 0 to 32 [45]. Prior research has used a similar approach to assess perceived social support and psychological impact among immigrant families [44, 46]. Cronbach’s α for the total scale was 0.96, indicating high internal consistency in our study population.

#### Sociodemographic and Acculturation Measures

Existing research on social support and social ties and their relationship to Asian American physical and mental health [16–18] informed our selection of relevant covariates. These included self-reported gender, age, marital status, employment status and education level. Participants also reported their length of residence in the U.S., spoken English proficiency, and Korean identity. Spoken English proficiency asked respondents to choose from five categories (fluent like a native speaker/well/not bad not good/poorly/not at all). Korean identity was measured using a single item adapted from the Suinn-Lew Asian Self-Identity Acculturation Scale [47], which offered five categories (very Korean/mostly Korean/bicultural/mostly westernized/very westernized) [48].

### Dependent Measures

#### Physical and Mental Health

Physical and mental health were measured by the English and Korean versions of the Short Form 12 Survey (SF-12), a shorter version of the SF-36 Health Survey Questionnaire, which generates two summary scores: the Physical Component Summary (PCS) and the Mental Component Summary (MCS) [49]. Raw scores were weighted using SF-12 reference values to allow comparison of specific groups of participants to general U.S. population norms [50]. The measures exhibited good reliability among our participants (MCS, *α* = 0.856; PCS *α* = 0.864).

### Analysis

Descriptive statistics summarized participant sociodemographic and psychosocial characteristics, social tie and social support scores, and baseline and follow-up physical and mental health outcomes (Table 1). Bivariate analyses explored associations between social ties and social support scores and cohort characteristics. Independent samples t-tests compared mean scores across categorical variables, while Pearson correlation coefficients quantified relationships between continuous variables and social ties and social support scores (Tables 2 and 3).

Tables 4 and 5 present multivariable linear regression analyses. Table 4 examines cross-sectional predictors of PCS and MCS health status at baseline. Table 5 presents factors associated with PCS and MCS scores at follow-up, follow-up health scores, controlling for baseline health (PCS or MCS scores). Two models are presented for each outcome: a full model including all sociodemographic and psychosocial factors as covariates, and a final most parsimonious model. To derive final models, forward stepwise selection was used was applied [51], retaining only predictors that contributed significantly (*p* < 0.05) to explaining variance in each outcome. Regression diagnostics, including tolerance and variance inflation factor, were examined to assess multicollinearity, which was found to be of no concern. All data cleaning and analyses were conducted using SPSS IBM statistics (Version 29) [52].

## Results

Initial medical record review identified 552 potentially eligible patients; 140 did not return for care during the pandemic, 20 were ineligible or declined screening, 27 refused to participate, and 2 did not complete surveys [40]. Of 363 participants completing baseline surveys, 318 (88%) completed follow-up surveys, 42 (11%) refused or were lost to follow-up, and three (1%) had died. Nine of the 318 were missing key variables for this analysis, yielding an analytical cohort of 309 participants.

### Characteristics of Study Participants

The univariate descriptors reported in Table 1, show that the majority were male (56.6%), aged 60–82 years (56.3%), married (79.9%), employed (63.4%), and had completed college or graduate training (57.7%). Most participants had lived in the U.S. for more than 30 years (59.2%), with an average length of residence of 32.5 years, with only 9 participants reporting being born in the U.S. In spite of the relatively long U.S. tenure, only 27.2% rated their English proficiency as fluent or well, and 57.9% saw themselves as very or mostly Korean. The average social ties score was 4.67 (SD = 1.3, range 0–6), and the average social support score was 21.70 (SD = 8.33, range 0–32). Social ties and social support at baseline were modestly but significantly positively correlated (*r* = 0.155, *p* = 0.006). Consistent with the cohort age and the potential physical and emotional burden associated with CHB, the average baseline PCS score was 48.56 (SD = 7.23, range 26–60), and the average baseline MCS score was 47.82 (SD = 6.87, range 21–60). Follow-up scores were similar, with the average PCS of 48.83 (SD = 7.55; range 16–59) and the average MCS of 48.33 (SD = 6.31, range 20–63). Baseline PCS and MCS scores were positively correlated (*r* = 0.164, *p* = 0.004), as were follow-up PCS and MCS scores (*r* = 0.303, *p* < 0.001).

### Association Between Cohort Characteristics and Social Ties

As shown in Table 2, higher average social ties scores were associated with being married (4.79 versus 4.15, *p* < 0.001), education of college or beyond (4.82 versus 4.47, *p* = 0.021), and identifying as bicultural or more Westernized (4.86 versus 4.54, *p* = 0.033). Social ties scores were positively correlated with length of residence in the U.S. (*r* = 0.187, *p* < 0.001), baseline PCS score (*r* = 0.163, *p* = 0.004), follow-up PCS score (*r* = 0.211, *p* < 0.001), and follow-up MCS score (*r* = 0.128, *p* = 0.025). However, social ties scores were not significantly correlated with age (*r* = 0.036, *p* = 0.530) or baseline MCS scores (*r* = 0.060, *p* = 0.294).

### Association Between Cohort Characteristics and Social Support

In Table 3, married participants reported significantly higher social support than those not married (22.74 versus 16.89, p<0.001), and currently employed participants reported higher social support than those not employed, with borderline statistical significance (22.41 versus 20.48, p=0.050). No significant differences in social support scores were observed by gender, college education, or Korean identity. Additionally, correlation analyses indicated a significant negative correlation between age and social support scores (r=−0.116, p=0.042), suggesting lower perceived social support among older participants. No significant correlation was found between the years living in the U.S. and social support (r=0.075, p=0.186). Social support was positively correlated with PCS scores (r=0.159, p=0.005) and MCS scores (r=0.195, p<0.001) at both baseline, and follow-up PCS score (r=0.151, p=0.008) and follow-up MCS score (r=0.216, p<0.001).

### Predictors of Baseline Physical and Mental Health

The final multivariable linear model in Table 4 identifies five significant predictors of baseline physical health and three significant predictors of baseline mental health. Older age (β=−0.292, *p* < 0.001) and being female (β=−0.158, *p* = 0.003) were associated with lower baseline PCS scores, while being married (β = 0.179, *p* < 0.001), being employed (β = 0.147, *p* = 0.009), and having a higher social ties score (β = 0.134, *p* = 0.009) were associated with higher baseline PCS scores.

Higher social support scores (β = 0.206, *p* < 0.001), older age (β = 0.265, *p* < 0.001), and being currently employed (β = 0.171, *p* = 0.004) were associated with higher baseline MCS scores. In contrast to the patterns observed for physical health, gender, marital status, and social ties scores were not significantly associated with mental health. Having a college education was also not significantly associated with either physical or mental health scores at baseline.

### Predictors of Follow-Up Physical and Mental Health

The final multivariable linear regression models in Table 5 build upon the findings in the previous models (Table 4) and examines covariates related to follow-up physical and mental health scores while controlling for baseline health scores.

#### Follow-up Physical Health Score

Self-assessed physical health at two-year follow-up is significantly and positively related to baseline physical health. After adjusting for baseline health, three social demographic variables are significantly associated with better follow-up physical health. Having a college degree or greater level of education is positively associated with better follow-up PCS scores (β=0.142, p<0.001). In contrast, older age (β=−0.115, p=0.011) and being female (β=−0.110, p=0.012) were associated with lower follow-up PCS scores. Marital status and employment status were not statistically significant and were not retained in the final model predicting follow-up PCS scores.

In addition, baseline social ties scores were positively associated with better physical health at follow-up (β=0.108, p=0.012), indicating that participants with greater social ties at baseline had better physical health outcomes at follow-up. Similar to the results of the cross-sectional baseline model reported in Table 4, social support scores were not significantly related to follow-up PCS scores.

#### Follow-up Mental Health Score

At follow-up, after controlling for baseline level of self-assessed mental health, having a college or greater education level was significantly positively associated with emotional well-being two years later, as measured by follow-up MCS score. In contrast to its negative relationship to physical health, older age was positively associated with follow-up MCS score. No other sociodemographic attributes were significantly associated with follow-up MCS scores.

Social support score at baseline was a significant independent predictor of better follow-up MCS scores (β=0.136, p<0.007); however, higher social ties score at baseline was non-significant. Thus, although social ties were a key predictor of follow-up PCS scores (β=0.108, p=0.012), social support emerged as more important for follow-up MCS scores.

## Discussion

This analysis examined the relationship of diverse sociodemographic and immigration-related factors to the social ties and social support of Korean American patients with CHB and then investigated associations with both physical and mental health. The results suggest that social ties and social support are important but distinct factors within this population, both in terms of their psychosocial correlates, as well as their role as markers of physical and mental health.

### Factors Associated with Greater Social Ties and Support

The bivariate findings highlight that several factors related to acculturation (more years of residence, a more “Western” identity, and self-rated English language fluency) are cross-sectionally associated with higher social ties scores. This suggests that among immigrant groups, language skills can enhance one’s ability to form and maintain social connections, which may not be the same for non-fluent speakers [53, 54]. Specifically, among Korean Americanswith CHB, English language proficiency may facilitate better communication not only with their U.S.-born children who may prefer English, but also with healthcare providers and the broader healthcare system. Previous research shows that immigrants who have limited English proficiency face major challenges when trying to access healthcare services, which results in reduced access to quality care and lower health literacy and ultimately leads to worse health outcomes and reduced social integration [55]. This underscores the importance of language skills in accessing and navigating these resources effectively.

However, these associations are cross-sectional and may best be interpreted as bi-directional. It is plausible that stronger ties offer opportunities to interact with more acculturated family and friends and strengthen an individual’s own language or cultural skills.

In addition, higher levels of formal education, and being married also predicted greater ties; however, cross-sectionally within this group, personal ties did not vary by gender, age, or work status. This finding suggests that interventions to strengthen social ties should broadly target those, across diverse gender and age groups, who are linguistically isolated or have lower education attainment. In contrast with social ties, social support does not vary significantly by Korean identity, years living in the U.S., or education level. Unlike social ties where employment status and age were not significantly associated, social support is lower among older participants, and conversely higher among those currently working. In addition to reporting greater social ties, married respondents also have significantly greater social support. English proficiency is a factor associated with both social ties and social support; participants with higher English proficiency fared better in both areas. These findings regarding the importance of marital status, language proficiency, and younger age as predictors of social support align with previous studies [54, 56].

### Associations with Physical and Psychological Well-Being Cross-Sectionally and Over Time

Our multivariable models allow comparison of both cross-sectional and temporal relationships, and consider how, within this specific population, social resources and relationships may shape physical and mental health functioning over time.

Cross-sectionally, after adjusting for age, both employment and marital status are correlates of physical health, suggesting that work and partner roles are markers of physical well-being among these persons living with CHB [54]. The significant link between work and both physical and mental health scores may reflect the unique challenges related to work for Korean immigrants living with CHB. Employment can provide not only economic security, but also social connections that are important for maintaining overall mental and physical health and well-being.

However, for immigrants who may work long hours in self-owned small businesses, the inability to continue physically demanding work also may be a marker for worse health, due to CHB or other chronic health issues. Previous research shows that loss of employment has been linked to declines in self-assessed health, but can affect groups differently based on ethnic background, immigration status, and social environment [57]. Moreover, immigrant workers need not only employment, but also workplace belonging and social connectedness to maintain their psychological well-being. Workplace demands related to acculturation, as well as social exclusion at work, can lead to stress, anxiety and negative mental health effects [58].

Another key finding is that older age is significantly associated with lower physical health, but better mental health, as measured by MCS and PCS scores. This finding contrastswith with findings that older age is associated with poorer self-rated mental health [17]. We might speculate even after immigration, within Korean Americancommunities and families, the traditional cultural value of revering and caring for older persons remains significant, and helps older persons avoid aging-related anxiety or depression [59].

Our multivariable findings suggest that both cross-sectionally and prospectively, greater social ties are linked to physical health, while higher social support aligns with improved mental health. This finding that greater social ties are associated with higher PCS scores among individuals with chronic illness, aligns with other work, which found that various social connections, including friends, are positively related to self-rated physical health [53]. This highlights the importance of social interactions in promoting physical health, possibly by encouraging healthier lifestyles through social obligations and expectations, as well as providing a connection to broader resources, such as information for better health management [53]. In addition, our results build on existing evidence regarding social support’s relationship with mental health [11, 54], suggesting that emotional and practical assistance from those closest to a person is key in maintaining good mental health, among individuals managing CHB [54] or other chronic diseases [11].

These findings align with a broader body of prior studies across multiple cultures and types of illnesses, which confirms the distinct contributions we observed, between different types of social resources and health outcomes. Soares et al. (2013), for example, studied long-term survivors of Hodgkin lymphoma and found that social network size and social support dimensions, especially affective and emotional support, were positively associated with both physical and mental health as well as reduced fatigue [60]. Their use of the SF-12 facilitates direct comparison, finding that physical health (PCS) depended on network structure, while mental health (MCS) depended on social support. Associations persisted even years after treatment, suggesting enduring health benefits of supportive interpersonal connections [60]. Their research underscores the relevance of disentangling network structure and support function when examining chronic disease outcomes.

A four-year longitudinal study of older adults in rural South Korea highlights the significance of consistent interpersonal interaction in preserving health-related quality of life over time [61]. This study specifically focused on “meeting with friends” which was highly linked to better physical and mental health outcomes, whereas discontinuing informal social connections was experienced as the greatest decline in both PCS and MCS, with the negative effects especially pronounced among women [61].

The importance of interpersonal ties in chronic disease management is further supported by Reeves et al. (2014), who argued that self-management is not an isolated behavior but a social process embedded in one’s network of care [63]. In their longitudinal study of patients with heart disease or diabetes in the United Kingdom, greater social involvement and help with illness management from network members were associated with better emotional health, improved coping, and reduced healthcare utilization [62]. However, unlike our findings, they reported that social network size did not directly predict improved physical health [62]. This contrast may be attributable to contextual differences: Reeve’s study’s participants had access to a national health system, possibly diminishing their reliance on social networks for physical health maintenance [62].

In contrast, Korean American immigrants with CHB may depend more heavily on close social ties for disease management due to limited access to linguistically or culturally appropriate healthcare. These differences highlight how the impact of social ties on health may be shaped by the broader social and structural context in which individuals live. Moreover, Reeves et al. (2014) noted that larger networks enhanced emotional health and self-management capacity, stating that “it did improve patients’ ability to cope with their condition(s), both practically and emotionally.” [62]. While our study found social ties to be more strongly linked to physical health, this echoes our broader conclusion: relational networks offer more than emotional support; they also serve as a functional resource for managing chronic conditions, particularly in underserved communities.

Taken together, our results highlight the need for improving the breadth and depth of social contacts by means of programs that support social engagement, diverse role integration, and emotional support to promote long-term health in aging immigrant populations confronting chronic disease. Our cross-sectional associations have utility in identifying subgroups within clinical populations who are at risk for reduced social resources. This can alert clinicians, social service and community groups, and patients’ families to prioritize these individuals for intervention. More importantly, the longitudinal findings confirm that the absence of these resources may actually contribute to declines in health over time.

### Strengths and limitations

The study has several strengths, including its focus on a specific population, the use of well-established measures for both social support and health outcomes, and the bilingual data collection process, which made it accessible to all participants. A key strength of this study is its use of a longitudinal design, which allowed us to examine the dynamic relationships between social ties, social support, and physical and mental health outcomes over several years.

The social convoy theoretical framework guided our distinction between structural (social ties) and functional (social support) dimensions of social relationships. This distinction, especially within the context of Korean American patients living with CHB, provides culturally grounded insights into how ties with family and close friends shape health outcomes in this vulnerable population.

Despite these strengths, this study has several limitations. First, we also chose to focus on interpersonal ties, rather than larger networks, because our experience in this population suggests that support for the very personal and potentially stigmatizing experience of CHB is less likely to come from casual network members such as coworkers. Our approach emphasized informal social ties over structured elements such as work or community volunteering, which could be less sensitive metrics for linguistically isolated, chronically ill immigrant populations such as ours. This means, however, that our findings are not directly comparable to the body of research using standard network analysis.

Although longitudinal, our findings cannot completely eliminate the possibility of selection bias. Healthier individuals are more able to build and maintain strong relationships across their entire lives, and our adjustment for baseline health status does not completely control for a possibly bidirectional effect between health and social connections.

Additionally, findings may not be generalizable to younger healthier second-generation Korean Americans or other Asian subgroups, as the sample focused primarily on older Korean Americanimmigrants with CHB. Finally, drawing our study participants from two culturally concordant clinical settings may limit generalizability to the experiences of other Asian American CHB patients.

### New Contribution to the Literature

The study adds specific insights into the Korean American-CHB patient experience, examining the role of social ties and social support separately in both physical and mental health status. Furthermore, it is among the few studies to examine how these social relationships predict follow-up physical and mental health outcomes over time, controlling for baseline health status.

This study also contributes to the broader theoretical discourse by suggesting that the utility of social ties in promoting health may vary significantly across different cultural contexts. For example, while many models [31] argue broadly that larger social ties correlate with better health outcomes, for Korean American CHB patients, the quality and cultural relevance of these ties may be more critical than their size. The significant associations we found between personal ties (e.g., family and close friends) and health outcomes support the notion that networks are more than just a set of relationships; they are integral to the provision of meaningful social ties. This suggests that future theoretical models should account for cultural differences in how social ties and support systems are structured and utilized.

Given the average age of 60, this cohort is likely to continue to experience age-related challenges. The mMOS-SS is designed to assess social support in a way that is particularly relevant for older adults, making it a suitable tool for this age group [63]. Moreover, the mMOS-SS could be further adapted to increase its cultural relevance, capturing the unique social support dynamics within the Korean American community, which often includes higher family ties and community support networks.

## Conclusion

This research demonstrates how social ties and social support function independently yet work together to shape health results. Healthcare providers and public health programs can enhance physical and psychological well-being in the CHB community through their understanding and utilization of existing social connections and support systems. The development of targeted interventions to strengthen social connections should focus on populations who face increased risks of social isolation including immigrants with chronic health challenges.

## Figures and Tables

**Table 1:** Demographic and Psychosocial Characteristics of Cohort (N=309)

Demographic Characteristics	*N* (%)
Gender	
Male	175 (56.6%)
Female	134 (43.4%)
Age, mean (± SD), range	59.64 (± 10.57),19–82
19–39	11 (3.6%)
40–59	124 (40.1%)
60–82	174 (56.3%)
Marital Status	
Married	247 (79.9%)
Living with a partner	7 (2.3%)
Separated	4 (1.3%)
Divorced	20 (6.5%)
Widowed	18 (5.8%)
Never Married	13 (4.2%)
Employment Status	
Student	4 (1.3%)
Employed	196 (63.4%)
Unemployed	20 (6.5%)
Retired	56 (18.1%)
Housewife	33 (10.7%)
Education level	
< high school	16 (5.2%)
High school graduate or GED +	66 (21.3%)
Some Colleges	48 (15.5%)
College graduate	117 (37.7%)
Graduate or professional School	62 (20.0%)
Spoken English Proficiency	
Fluent like a native speaker	37 (12.0%)
Well	47 (15.2%)
Not bad not good	124 (40.1%)
Poorly	89 (28.8%)
Not at all	12 (3.9%)
Korean/Western Identity Index	
Very Korean	97 (31.4%)
Mostly Korean	82 (26.5%)
Bicultural	108 (35.0%)
Mostly Westernized	15 (4.9%)
Very Westernized	7 (2.3%)
Years living in the US, mean (± SD), range	32.51 (± 10.93), 5–58
< 10 years	3 (1.0%)
10–30 years	114 (36.9%)
> 30 years	183 (59.2%)
Born in the US	9 (2.9%)
Psychosocial Characteristics - Index and Possible Scores	**Mean (± SD)**, **range**
Social Ties Score (Baseline)	4.67 (± 1.32), 0–6
Social Support Score (Baseline)	21.70 (± 8.33), 0–32
Baseline Physical Health Score (0–100)	48.56 (± 7.23), 26–60
Baseline Mental Health Score (0–100)	47.82 (± 6.87), 21–60
Follow-Up Physical Health Score (0–100)	48.83 (± 7.55), 16–59
Follow-Up Mental Health Score (MCS) (0–100)	48.33 (± 6.31), 20–63
Bivariate Correlations	**Correlations(*p*-value)**
Social Ties and Social Support Scores	0.155 (0.006)
Baseline PCS and MCS	0.164 (0.004)
Follow-up PCS and MCS	0.303 (< 0.001)

**Table 2:** Bivariate associations between cohort characteristics and baseline social ties scores (*N* = 309)

Characteristics	Mean (± SD)	*p*-value
Gender		0.782
Male	4.69 (1.36)	
Female	4.65 (1.29)	
Education level		0.021
Less than College Graduate	4.47 (1.41)	
College Graduate or Beyond	4.82 (1.24)	
Employment Status		0.589
Not Working	4.62 (1.29)	
Currently Working	4.70 (1.35)	
Marital Status		< 0.001
Not Married	4.15 (1.46)	
Currently Married	4.79 (1.27)	
English Proficiency		0.112
Not Fluent	4.60 (1.36)	
Fluent	4.87 (1.22)	
Korean Identity (Western Identity Index)		0.033
Very/Mostly Korean	4.54 (1.40)	
Bicultural/Mostly/Very Westernized	4.86 (1.19)	
Bivariate Correlations with Social Ties Score	Correlations	*p*-value
Age	0.036	0.530
Years living in the US	0.187	< 0.001
Baseline Physical Health Score	0.163	0.004
Baseline Mental Health Score	0.060	0.294
Follow-up Physical Health Score	0.211	< 0.001
Follow-up Mental Health Score	0.128	0.025

**Table 3:** Bivariate associations between cohort characteristics and baseline social support scores (*N* = 309)

Characteristics	Mean (± SD)	*p*-value
Gender		0.109
Male	22.35 (8.79)	
Female	20.85 (7.63)	
Education level		0.345
Less than College Graduate	21.18 (8.31)	
College Graduate or Beyond	22.08 (8.34)	
Employment Status		0.050
Not Working	20.48 (8.15)	
Currently Working (Employed)	22.41 (8.37)	
Marital Status		< 0.001
Not Married	16.89 (8.13)	
Currently Married	22.74 (8.01)	
Self-Rated English Proficiency		0.051
Not Fluent	21.14 (8.28)	
Fluent	23.21 (8.32)	
Korean Identity (Western Identity Index)		0.425
Very/Mostly Korean	21.36 (8.65)	
Bicultural/Mostly/Very Westernized	22.15 (7.87)	
Bivariate Correlations with Social Support Score	Correlations	*p*-value
Age	−0.116	0.042
Years living in the US	0.075	0.186
Baseline Physical Health Score	0.159	0.005
Baseline Mental Health Score	0.195	< 0.001
Follow-up Physical Health Score	0.151	0.008
Follow-up Mental Health Score	0.216	< 0.001

**Table 4:** Multivariable linear regression Models, predicting participants’ baseline physical health component summary score (PCS) and mental health component summary score (MCS) (*N* = 309)

	Baseline Physical Component Summary Score (PCS)^a^						Baseline Mental Component Summary Score (MCS)^b^					
	Full Model			Reduced Model			Full Model			Reduced Model		
Predictor variables		Standardized			Standardized			Standardized			Standardized	
	*B* (SE)	b	*p*-value	*B* (SE)	b	*p*-value	*B* (SE)	b	p-value	*B* (SE)	b	*p*-value
Social Ties Score	0.654 (0.281)	0.120	0.020	0.729 (0.277)	0.134	0.009	0.074 (0.293)	0.014	0.801			
Social Support Score	0.025 (0.046)	0.029	0.585				0.164 (0.048)	0.199	<0.001	0.170 (0.045)	0.206	<0.001
Age (in years)	−0.189 (0.038)	−0.276	<0.001	−0.200 (0.037)	−0.292	<0.001	0.168 (0.039)	0.257	<0.001	0.173 (0.038)	0.265	<0.001
Gender (1 = Female)	−2.165 (0.761)	−0.149	0.005	−2.303 (0.757)	−0.158	0.003	−0.241 (0.79)	−0.017	0.762			
Education (1 = College graduate+)	1.187 (0.748)	0.081	0.114				−0.335 (0.781)	−0.024	0.668			
Marital Status (1 = Married)	3.208 (1.008)	0.170	0.002	3.370 (0.978)	0.179	<0.001	0.346 (1.051)	0.019	0.742			
Employment (1 = Currently employed)	2.171 (0.840)	0.145	0.010	2.210 (0.840)	0.147	0.009	2.332 (0.876)	0.164	0.008	2.43 (0.83)	0.171	0.004

B unstandardized coefficient, SE standard error, b standardized coefficient

a. R^^2^^ = 0.256 (full), *R*^*^2^*^ = 0.250 (reduced), the reduced model included only predictors that were significant (*p* < 0.05)

b. R^^2^^ = 0.105 (full); *R*^*^2^*^ = 0.104 (reduced), the reduced model included only predictors that were significant (*p* < 0.05)

**Table 5:** Multivariable linear regression Models, predicting participants’ Follow-up physical health component summary score (PCS) and mental health component summary score (MCS), controlling for baseline health score (*N* = 309)

	Follow-up Physical Component Summary Score (PCS)^a^						Follow-up Mental Component Summary Score (MCS)^b^					
	Full Model			Reduced Model			Full Model			Reduced Model		
Predictor variables		Standardized			Standardized			Standardized			Standardized	
	B (SE)	b	p-value	B (SE)	b	*p*-value	B (SE)	b	p-value	B (SE)	b	*p*-value
PCS (baseline control)	0.546 (0.051)	0.523	<0.001	0.551 (0.049)	0.528	<0.001						
MCS (baseline control)							0.398 (0.047)	0.434	<0.001	0.401 (0.047)	0.437	<0.001
Social Ties Score	0.600 (0.250)	0.105	0.017	0.618 (0.245)	0.108	0.012	0.301 (0.240)	0.063	0.210			
Social Support Score	0.017 (0.040)	0.019	0.665				0.106 (0.040)	0.140	0.008	0.103 (0.038)	0.136	0.007
Age (in years)	−0.078 (0.035)	−0.109	0.024	−0.082 (0.032)	−0.115	0.011	0.081 (0.033)	0.136	0.015	0.073 (0.030)	0.122	0.017
Gender (1 = Female)	−1.611 (0.680)	−0.106	0.019	−1.675 (0.660)	−0.110	0.012	0.325 (0.652)	0.026	0.618			
Education (1 = College graduate+)	2.205 (0.663)	0.145	<0.001	2.207 (0.660)	0.142	<0.001	1.937 (0.641)	0.152	0.003	2.006 (0.629)	0.157	0.002
Marital Status (1 = Married)	0.048 (0.904)	0.002	0.958				−0.783 (0.863)	−0.048	0.365			
Employment (1 = Currently employed)	0.225 (0.749)	0.014	0.764				0.517 (0.727)	0.040	0.478			

B unstandardized coefficient, SE standard error, b standardized coefficient, Baseline control is baseline PCS for the follow-up PCS outcomes and baseline MCS for the follow-up MCS outcomes

a. *R*^^2^^ = 0.470 (full) *R*^^2^^ =0.470 (reduced), the reduced model included only predictors that were significant (*p* < 0.05)

b. *R*^^2^^ = 0.286 (full), *R*^^2^^ = 0.280 (reduced), the reduced model included only predictors that were significant (*p* < 0.05)

## Data Availability

Data are not publicly available due to the protected health information (PHI) involved, but de-identified data may be available in the future to researchers on request.

## Abbreviations

HBV: Hepatitis B Virus Infection
CHB: Chronic Hepatitis B
PCS: Physical Component Summary
MCS: Mental Component Summary
SNI: Social Network Index
SF12: Short Form 12 Survey
